# Neuroprotective effects of 3,5-di-o-caffeoylquinic acid *in vitro* and *in vivo*

**DOI:** 10.1186/1753-6561-5-S8-P20

**Published:** 2011-11-22

**Authors:** Junkyu Han, Hiroko Isoda

**Affiliations:** 1 Graduate School of Life and Environmental Sciences, University of Tsukuba. Tsukuba, Ibaraki 305-8572, Japan; 2 Alliance for Research on North Africa (ARENA), University of Tsukuba. Tsukuba, Ibaraki 305-8572, Japan

## Background

Caffeoylquinic acid (CQA) derivatives are natural functional compounds isolated from a variety of plants and possess a broad range of pharmacological properties, including antioxidant, hepatoprotectant, antibacterial, antihistaminic, anticancer, and other biological effects [1]. Recently, it has been demonstrated that CQA derivatives possess neuroprotective effects in Aβ-induced PC12 cell toxicity and in tetrahydropapaveroline (THP)-induced C6 glioma cell death [2]. One of the animal models that is used to study AD and aging is the senescence-accelerated mouse (SAM). The SAM model was developed in 1981, which originally consisted of nine major senescence-accelerated-prone mice (SAMP) substrains and three major senescence-accelerated-resistant mice (SAMR) substrains, each of which exhibits the characteristic disorders.

## Methodology

As *in vitro* experiment, the human neuroblastoma clonal SH-SY5Y cell were maintained at 37°C under 5% CO_2_ / 95% air. As *in vivo* experiment, the CQA-treated mice were orally administered with 3,5-di-O-CQA mixed with drinking water (6.7 mg/kg · day) for 1 month using oral administration tube and syringe. Proteomics analysis, real-time PCR, measurement of intracellular ATP content, Moris water maze were carried out to investigate the neuroprotective effect of CQA.

## Results

3,5-di-O-CQA had neuroprotective effect on Aβ_1–42_ treated cells. The mRNA expression of glycolytic enzyme (phosphoglycerate kinase-1; PGK1) and intracellular ATP level were increased in CQA treated SH-SY5Y cells. We also found that CQA administration induced the improvement of spatial learning and memory on SAMP8 mice, and the overexpression of PGK1 mRNA.

## Conclusion

CQA has a neuroprotective effect on Aβ_1–42_ treated SH-SY5Y cells. The mRNA expression of glycolytic enzyme (PGK1) and the intracellular ATP level were increased in CQA-treated SH-SY5Y cells. We also found that CQA administration induced the improvement of spatial learning and memory on SAMP8 mice, and the overexpression of PGK1 mRNA level. These findings suggest that CQA has a neuroprotective effect through the induction of PGK1 expression and ATP production activation.

**Figure 1:**
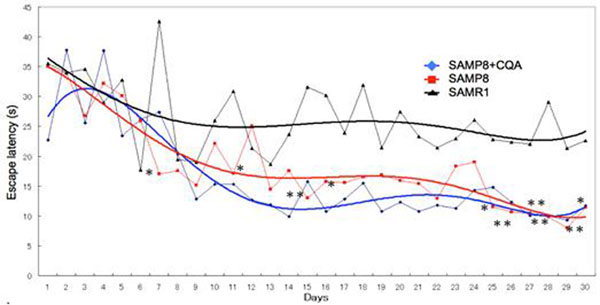
Effect of CQA on the spatial learning and memory of SAMP8 mice in MWM.
